# Digital three-dimensional planning combined with 3D-printed patient-specific guide-assisted step-cut osteotomy for correction of old cubitus varus deformity: a case report

**DOI:** 10.3389/fsurg.2026.1926289

**Published:** 2026-09-22

**Authors:** Xiaofan Huang, Fulin Tang, Jialin Zhuang, Weikun Li

**Affiliations:** Department of Upper Limb, Mianyang Orthopaedic Hospital, Mianyang, Sichuan, China

**Keywords:** 3D printed patient-specific guide, Cubitus varus deformity, mirror digital planning, posttraumatic malunion, step-cut translation osteotomy

## Abstract

Cubitus varus is the most common long-term complication following supracondylar humeral fractures. Although traditional step-cut osteotomy yields reliable clinical outcomes, the intraoperative osteotomy direction and angle remain largely dependent on the surgeon's experience, resulting in suboptimal precision. Although digital three-dimensional (3D) planning and 3D-printed patient-specific guides have been shown to improve osteotomy accuracy, their application in adolescent patients with long-standing cubitus varus deformity has rarely been reported. We report an 18-year-old male patient who developed progressive cubitus varus deformity 13 years after conservative treatment of a right supracondylar humeral fracture. Preoperatively, the carrying angle was −19°, with mildly restricted elbow motion and no ulnar nerve symptoms. Using mirror-image-based 3D digital planning, we designed and 3D-printed a patient-specific step-cut osteotomy guide and performed corrective osteotomy with lateral locking plate fixation. Intraoperatively, the guide was stably apposed to the bone surface, enabling precise execution of the osteotomy without displacement or interference. At 12-month follow-up, both the carrying angle and humeral-elbow-wrist angle had improved from −19° to 6.5°, elbow flexion-extension range improved from 5°–120° to 0°–135°, and the Mayo Elbow Performance Score was 95. No complications such as nonunion or nerve injury occurred, and the patient was satisfied with the surgical outcome. The combination of digital 3D planning and 3D-printed guide-assisted step-cut osteotomy may significantly enhance osteotomy accuracy and surgical safety, offering an effective approach for treating long-standing cubitus varus in adolescents. Nevertheless, given that this is a single-case report, broader studies are warranted to confirm its advantages.

## Introduction

Supracondylar humeral fractures are the most prevalent pediatric elbow fractures, comprising approximately 50%–70% of all such injuries (1). Despite standard treatment, cubitus varus remains the most common long-term complication, with a reported incidence of 10%–50% (2). It not only affects cosmesis but may also alter elbow biomechanics, restrict range of motion, and cause late-onset ulnar nerve palsy (3).

For patients with significant deformity and functional impairment, surgical correction is the recommended treatment (4). Although traditional step-cut osteotomy remains a reliable classic procedure for correcting cubitus varus, the intraoperative osteotomy position, direction, and angle are largely determined by the surgeon's visual estimation and experience. This reliance may result in under- or over-correction, suboptimal apposition of osteotomy surfaces, and internal fixation failure (5).

Recently, 3D-CT–based computer-assisted planning and 3D-printed patient-specific guides have been widely applied in deformity correction, significantly improving osteotomy accuracy and surgical safety (6). They have also been used in cubitus varus correction with favorable early results (7).

However, previously reported 3D-printed guide-assisted techniques for cubitus varus still have limitations: (1) guides are typically designed based solely on local bone surface morphology matching, lacking multi-point locking mechanisms anchored to anatomical reference points, which may lead to intraoperative displacement; (2) osteotomy designs are often simple planar cuts that do not exploit the mechanical interlocking of step-cut surfaces for rotational stability; and (3) reports on complex cases of long-standing deformities in adolescents (duration exceeding five years) remain scarce (8).

Compared with previously reported 3D-printed guide-assisted techniques, our method offers three technical advantages (9). First, digital mirror-image superimposition defines the three-dimensional deformity, including rotational components, in a single step, avoiding the measurement errors of two-dimensional radiographs (10). Second, the step-cut osteotomy guide pre-sets the position, direction, width, and depth of the osteotomy, ensuring that intraoperative cuts precisely correspond to the preoperative plan and reducing reliance on intraoperative subjective judgment (11). Third, the guide incorporates a multi-point locking structure that uses bony landmarks—the medial epicondyle tip, lateral epicondyle tip, and inferior margin of the olecranon fossa—to achieve stable placement and reliable apposition intraoperatively (12).

Herein, we report a case of old cubitus varus deformity in an adolescent successfully treated with digital 3D planning and 3D-printed guide-assisted step-cut osteotomy, and describe the design principles, surgical technique, and clinical outcomes to provide a reference for clinical practice (8).

### Case report

An 18-year-old male presented with a 13-year history of right cubitus varus deformity that had progressively worsened over the past year, accompanied by limited elbow motion. Thirteen years prior, the patient sustained a right supracondylar humeral fracture and was conservatively managed with closed reduction and cast immobilization at another institution. Mild cubitus varus was noted after fracture healing but was left untreated; over the past year, the deformity worsened and caused pain and discomfort during elbow flexion and extension. He reported no neurological symptoms (numbness or weakness in the hand), chronic illnesses such as hypertension or diabetes, smoking, alcohol consumption, or other previous surgeries or trauma.

### Physical examination

The patient was alert and cooperative with stable vital signs. The right elbow exhibited a prominent varus deformity, with a carrying angle of −19° compared with 6° on the contralateral side. No swelling or focal tenderness was observed. Active right elbow range of motion was flexion 120°, extension 5°, forearm pronation 70°, and supination 90°; the contralateral elbow had flexion 140°, extension 0°, and forearm rotation 85°–95°. Neurovascular examination revealed normal sensation and motor function of the right hand, no ulnar nerve symptoms, negative Tinel sign, a palpable radial pulse, and good peripheral perfusion. Muscle strength of the right upper extremity was 5/5 in all group. All physical examination data and images were obtained with written informed consent from the patients and their families. This study received an official waiver of ethical review from the Ethics Committee of Mianyang Orthopedic Hospital, with exemption approval number (2026-003-01). (Figure 1a).

**Figure 1:**
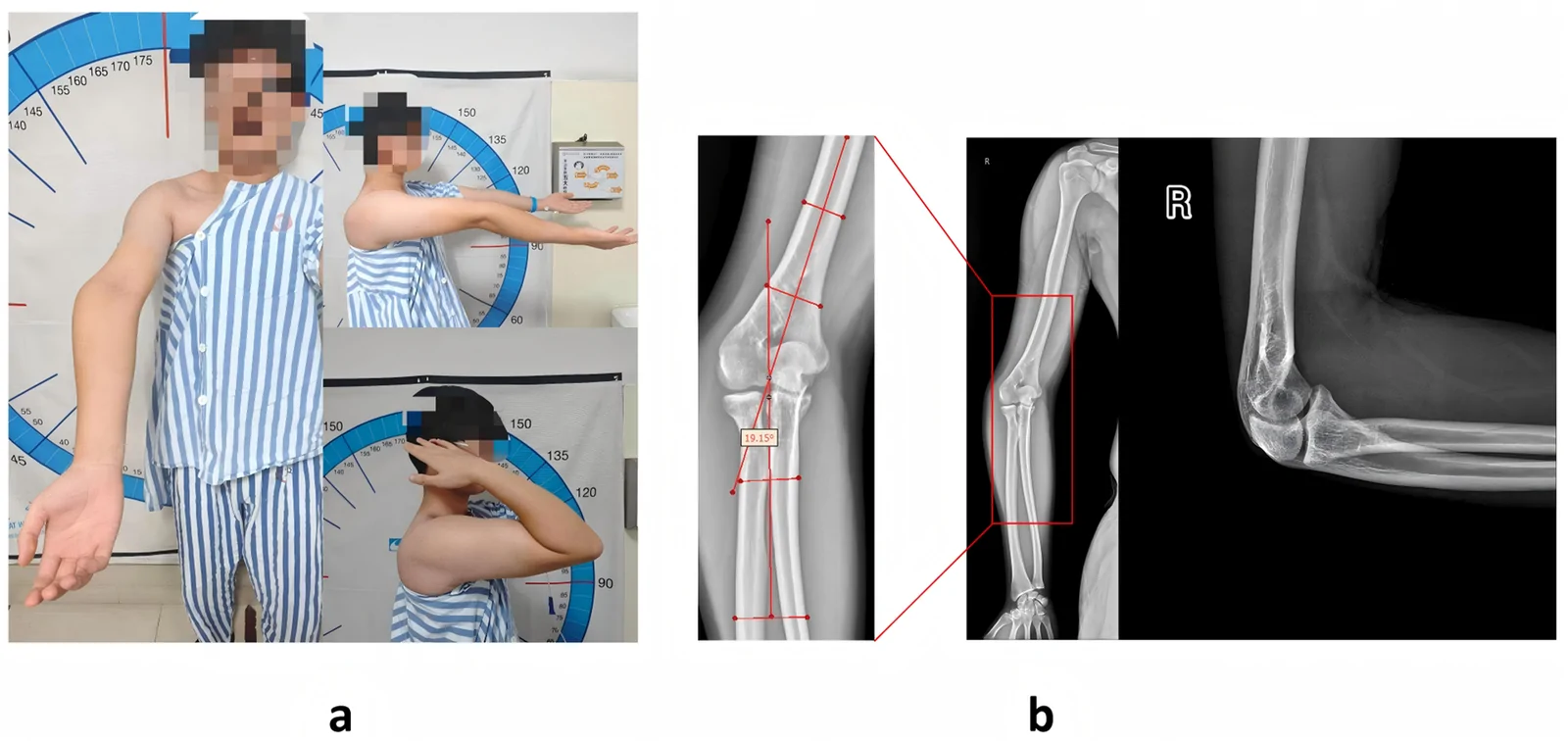
**(a)** Preoperative appearance and function of both upper extremities; **(b)** preoperative radiograph confirming right cubitus varus deformity with a carrying angle of −19°.

### Imaging findings

Preoperative anteroposterior radiographs showed a malunited old supracondylar humeral fracture with medial angulation of the distal humerus and a humeral-elbow-wrist angle (HEWA) of −19°; the lateral view demonstrated a normal distal humerus–olecranon relationship without degenerative changes (Figure 1b). Preoperative CT was performed using a 64-slice spiral scanner (slice thickness 0.625 mm, reconstruction matrix 512 × 512). Three-dimensional reconstruction revealed coronal varus deformity of the right distal humerus, with no rotational deformity, an intact articular surface, and no osteoarthritic changes (Figure 2).

**Figure 2:**
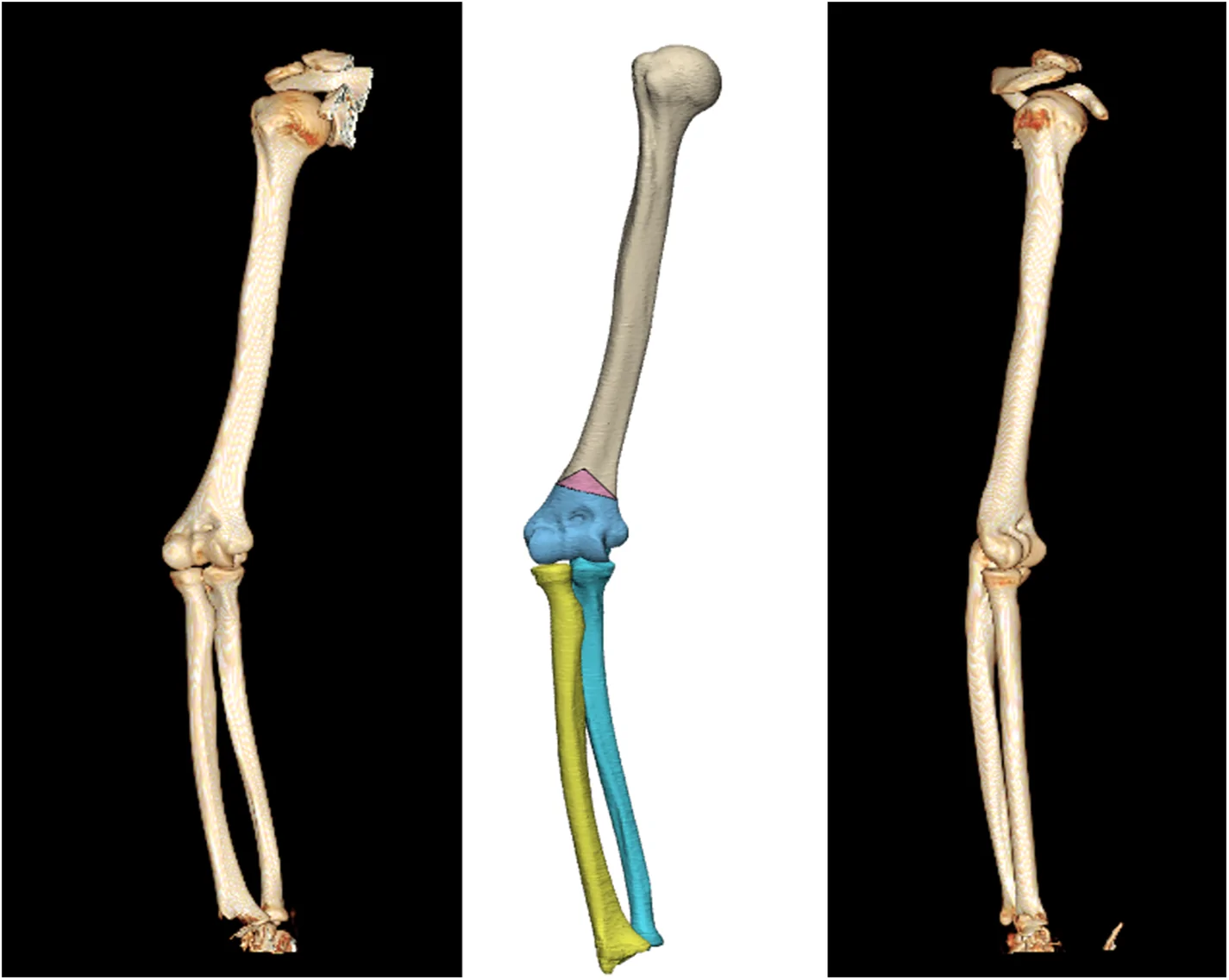
3d reconstruction showed a coronal varus deformity of the right distal humerus without rotational or sagittal deformity; the pink triangle indicates the osteotomy area.

### Preoperative digital planning and 3d-printed guide fabrication

The thin-slice CT data of the affected upper extremity were imported into medical 3D reconstruction software to construct a three-dimensional anatomical model of the distal humerus and elbow joint. The osteotomy was planned using the step translational osteotomy method described by Kim et al. (13). With the contralateral elbow as a mirror reference and a target humeral-elbow-wrist angle of 6°, the required correction angle was calculated as the sum of the varus angle and the target angle (angle B = 25°). The osteotomy plane was positioned 1 cm proximal to the olecranon fossa, and the intersection of its horizontal line with the medial cortex of the distal humerus was designated point B; this point served as an intraoperative reference, with a perpendicular distance of 3.9 cm to the elbow joint line. A line BC perpendicular to the humeral axis was drawn through point B. Point A was then determined so that the apex angle at A equaled the angle between the lateral cortex of the distal humerus and BC (angle A = 105°); with angle B (25°), point A was established. Triangle ABC represented the osteotomy region, with side lengths BC = 37 mm, AB = 27 mm, and AC = 19 mm. Since the distal humeral anterior tilt angle measured 38°, no sagittal plane correction was required (Figure 3a). A custom three-dimensional (3D)-printed osteotomy guide with prefabricated step-cut slots was fabricated using medical photosensitive resin (Figures 3b,c).

**Figure 3:**
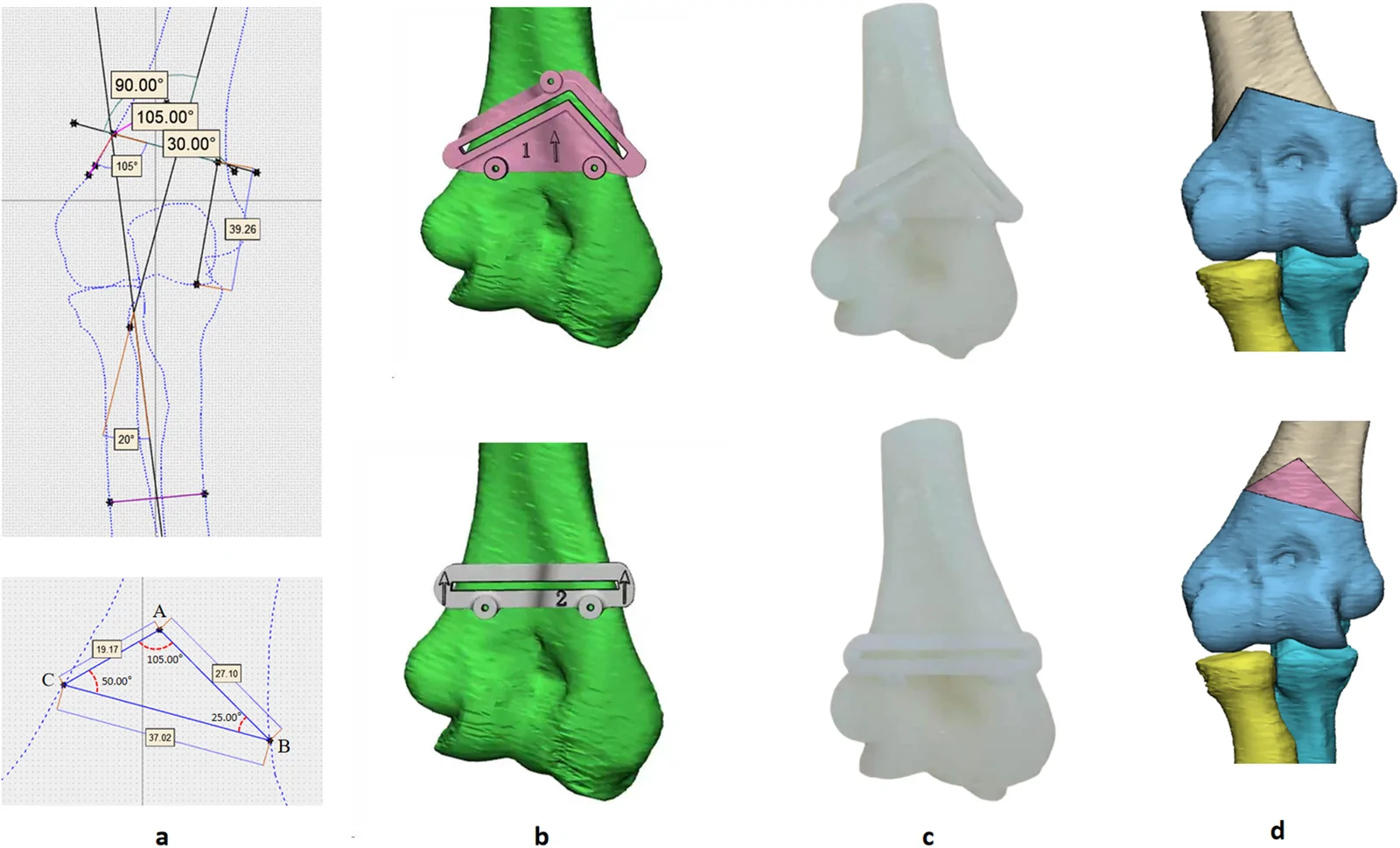
Design of the patient-specific 3D-printed surgical guide: **(a)** osteotomy planning using the contralateral elbow as a mirror reference: With the unaffected elbow as a mirror reference, the contralateral humerus–elbow–wrist angle of 6° was set as the correction target. The required osteotomy angle was calculated by adding the varus angle (angle B, 25°) to the target angle. First, the medial osteotomy point B was identified: the osteotomy plane was 1 cm proximal to the olecranon fossa, and its horizontal line intersected the medial cortex of the distal humerus at point B (as an intraoperative reference, the vertical distance from point B to the elbow joint line was 39.26 mm). A line perpendicular to the humeral axis was then drawn through point B, intersecting the lateral cortex of the distal humerus at point C. Point A was determined such that the apex angle equaled the angle between the lateral cortex of the distal humerus and BC (angle A = 105°); therefore, point A was defined by angle B = 25° and angle A = 105°; **(b)** design of a model with a prefabricated stepped cutting slot based on 3D reconstruction; **(c)** fabrication of the customized 3D-printed osteotomy guide with the prefabricated stepped slot using medical photosensitive resin; **(d)** 3-D reconstructed images after simulated osteotomy for preoperative planning.

### Surgical technique

Under general anesthesia, the patient was placed in the lateral decubitus position with a tourniquet on the right upper arm. After routine sterilization and draping, an approximately 18 cm posterior curvilinear incision was made over the elbow. The skin and subcutaneous tissue were incised, the ulnar nerve was carefully dissected and protected within the cubital tunnel, and the interval between the medial and lateral heads of the triceps was developed. Subperiosteal dissection exposed the supracondylar osteotomy region of the humerus.

The 3D-printed patient-specific osteotomy guide (Guide 1) was applied to the posterior surface of the distal humerus. After confirming complete apposition, Kirschner wires were inserted through the positioning holes for temporary fixation. Fluoroscopy confirmed that the perpendicular distance from the medial osteotomy point B to the elbow joint line was approximately 3.9 cm. Using the preset guiding slot, an oscillating saw completed the osteotomy layer by layer. Guide 2 was then applied to confirm points B and C, and the transverse osteotomy was completed, with care taken to protect surrounding neurovascular structures (Figure 4).

**Figure 4:**
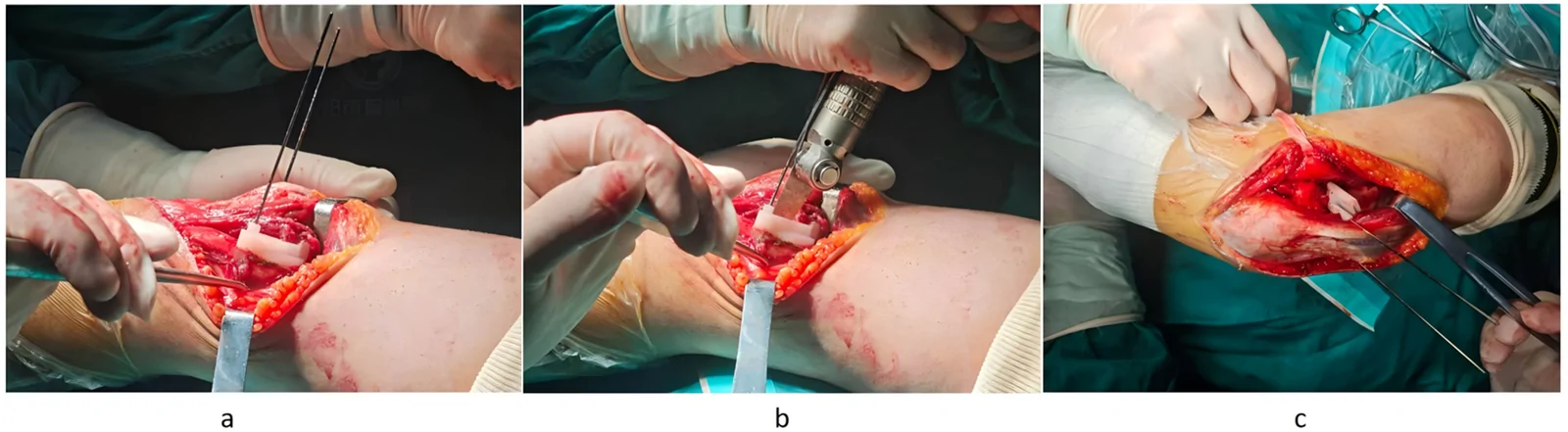
Intraoperative application of the patient-specific 3D-printed osteotomy guides (guide 1): **(a)** guide 1 was placed on the posterior surface of the distal humerus, kirschner wires were inserted through the positioning holes for temporary fixation; **(b)** using the preset guiding slot, osteotomy was completed stepwise with an oscillating saw; **(c)** intraoperative photograph after completion of the A-C and A-B osteotomies using guide 1.

After completion of the osteotomy, the distal fragment was rotated laterally and translated medially according to the preoperative plan, allowing points A and C to coincide and the step-cut bone cortices to interdigitate, thereby correcting coronal alignment of the elbow. Temporary fixation was achieved with Kirschner wires, and fluoroscopy confirmed restoration of the carrying angle to 5°–10°. Anatomical locking plates were then applied to the medial and lateral aspects of the distal humerus with screws for rigid fixation. Final fluoroscopy verified satisfactory osteotomy position, correction angle, and implant placement. The surgical field was irrigated, meticulous hemostasis was achieved, and the ulnar nerve was transposed anteriorly into a subcutaneous soft tissue bed. The incision was closed in layers with one plasma drainage tube placed posterolaterally. Sterile dressings were applied with compression, and the elbow was immobilized with a sling. Intraoperative blood loss was approximately 200 mL, fluoroscopy was performed four times, and the operative time was 2 hours and 10 minutes.

### Postoperative management

Postoperatively, the patient received anti-edema, analgesic, and prophylactic antibiotic therapy, with close monitoring of distal perfusion, sensation, and motor function. The drainage tube was removed on postoperative day 2 (total output 30 mL). At 2 weeks, the incision had healed by primary intention; sutures were removed, and passive elbow flexion-extension exercises were initiated. At 4 weeks, active flexion-extension and forearm rotation training were gradually introduced, avoiding premature weight-bearing and forceful activities. Light daily upper extremity activities were permitted at 8 weeks, and the patient resumed pre-injury work and sports activities at 12 weeks.

### Follow-up results

Immediate postoperative imaging demonstrated correction of the humeral-elbow-wrist angle to 7° (contralateral 6°), with preservation of the distal humeral anterior tilt angle of 38°. The step-cut osteotomy surfaces were well-opposed and interdigitated, without secondary angulation or S-shaped deformity (Supplementary Image 1a).

At the 12-month final follow-up, the carrying angle was 6° and the Mayo Elbow Performance Score was 95. Active elbow flexion-extension range was 0°–135° (contralateral 0°–140°), with forearm pronation 85° and supination 90° (contralateral 85° and 90°, respectively). Preoperative soreness and fatigue after weight-bearing labor had completely resolved. Ulnar nerve sensation and motor function were normal, without muscle atrophy. The elbow appearance was symmetric, and patient satisfaction was high (Supplementary Image 1b). The patient recently underwent removal of internal fixation, and elbow function has been fully restored to the pre-injury level (Supplementary Image 1c,d). No complications such as infection, nonunion, implant failure, nerve injury, or heterotopic ossification occurred throughout the treatment period. Table 1 presents the physical examination findings of the elbow joint at each designated time point.

**Table 1:** Comparison of elbow parameters between affected and healthy sides.

Parameter	Affected (Right) Preoperative	Affected (Right) Postoperative 12 months	Healthy (Left) Reference
Carrying angle (°)	−19°	6°	6°
HEWA (°)	−19°	6.5°	6°
Elbow flexion-extension range (°)	5°–120°	0°–135°	0°–140°
Forearm rotation range (°)	70°–90°	85°–90°	85°–90°
Mayo Elbow Performance Score	70	95	100

## Discussion

The principal goals of correcting cubitus varus are to restore the normal carrying angle, re-establish the biomechanical axis of the elbow, and prevent complications such as nerve injury and nonunion. Although traditional step-cut osteotomy is widely used, its accuracy depends heavily on the surgeon's experience and subjective judgment, with reported inadequate correction rates of 10%–25% (14). This largely stems from difficulty in precisely controlling the osteotomy position, direction, and angle intraoperatively, particularly in complex deformities with rotational components or articular surface tilting (15).

Digital 3D planning and 3D-printed guides offer an effective solution (16). Using mirror-image technology to map the contralateral healthy side to the affected side, three-dimensional deformity parameters can be precisely quantified preoperatively, and the optimal osteotomy plan determined through computer simulation (13). Patient-specific guides then transfer this plan to the operating room, allowing the osteotomy to precisely match the preoperative design and thereby significantly improving accuracy (17).

Our technique differs from previously reported 3D-printed guide-assisted methods in three key respects. First, the guide employs a three-point locking structure involving the medial epicondyle, lateral epicondyle, and olecranon fossa, providing a larger bone contact area than simple surface-matching guides and thereby improving intraoperative fixation reliability. Second, the step width and depth were quantitatively determined preoperatively from bilateral CT data (width 12 mm, depth 4 mm), ensuring maximal contact area and interdigitation of the osteotomy surfaces without bone grafting, thereby achieving immediate mechanical stability. Third, the step cross-section was digitally matched to provide rotational and angular stability, a feature not explicitly described previously. These technical features make our technique more reproducible and potentially more accurate than conventional 3D-printed guide surgery, although larger multicenter studies are needed to validate this conclusion.

Biomechanically, the step-cut osteotomy provides a larger contact area and inherent rotational stability, offering a mechanical basis for early functional rehabilitation (18). Locking compression plates have also demonstrated favorable bending and torsional resistance in distal humeral osteotomy fixation, providing sufficient initial stability to support early rehabilitation (19).

However, the delayed union rate (5.9%) and nerve palsy rate (5.9%) reported for traditional step-cut osteotomy are derived from larger cohort studies, whereas the present study is a single case report and does not permit statistical comparison (20). The potential advantages of our technique over traditional methods therefore lie mainly in precise osteotomy execution and the initial rotational stability conferred by the step-cut design, which require confirmation through prospective comparative studies with larger samples (18).

This study has several limitations: (1) it is a single case report with a small sample size, precluding statistical analysis; (2) the follow-up duration was only 12 months, and longer-term observation is needed to confirm maintenance of correction; (3) the technique requires 3D-CT scanning, specialized software design, and 3D printing equipment, incurring additional costs and equipment requirements; and (4) the incorporation of soft tissue tension effects on osteotomy site apposition into the planning process requires further investigation (21).

## Conclusion

Digital 3D planning combined with 3D-printed guide-assisted step-cut osteotomy is an effective approach for treating long-standing cubitus varus deformity in adolescents. It enables precise correction, provides immediate rotational stability, and yields satisfactory clinical outcomes with a low complication rate. However, as a single case report, its superiority requires validation in larger prospective studies. This technique offers a novel and feasible approach for the precise treatment of complex cubitus varus deformities.

## Data Availability

The original contributions presented in the study are included in the article/Supplementary Material, further inquiries can be directed to the corresponding author.
